# Neurological and Sleep Disturbances in Bronchiectasis

**DOI:** 10.3390/jcm6120114

**Published:** 2017-11-30

**Authors:** Chun Seng Phua, Tissa Wijeratne, Conroy Wong, Lata Jayaram

**Affiliations:** 1 Department of Medicine, Melbourne Clinical School, University of Melbourne, Melbourne, VIC 3010, Australia; chunsengphua@yahoo.com (C.S.P.); twi@unimelb.edu.au (T.W.); 2 Department of Neurology, Western Health, St. Albans, VIC 3021, Australia; 3 Department of Medicine, Faculty of Medicine, University of Rajarata, Saliyapura AD 50008, Sri Lanka; 4 Department of Psychology and Counselling, School of Psychology and Public Health, College of Science, Health and Engineering, La Trobe University, Melbourne, VIC 3086, Australia; 5 Department of Respiratory Medicine, Middlemore Hospital, Auckland 2025, New Zealand; conroy.wong@middlemore.co.nz; 6 Department of Respiratory and Sleep Medicine, Western Health, St. Albans, VIC 3021, Australia

**Keywords:** bronchiectasis, sleep, stroke, cognitive, depression, anxiety, infection, aspiration, cerebral, psychological

## Abstract

Bronchiectasis unrelated to cystic fibrosis is a chronic lung disease that is increasingly recognised worldwide. While other common chronic lung conditions such as chronic obstructive lung disease have been associated with cardiovascular disease, there is a paucity of data on the relationship between bronchiectasis and cardiovascular risks such as stroke and sleep disturbance. Furthermore, it is unclear whether other neuropsychological aspects are affected, such as cognition, cerebral infection, anxiety and depression. In this review, we aim to highlight neurological and sleep issues in relation to bronchiectasis and their importance to patient care.

## 1. Introduction

Bronchiectasis is a chronic lung disease characterised by repeated episodes of infection and chronic inflammation that can lead to significant morbidity and mortality [1,2]. Bronchiectasis unrelated to cystic fibrosis, hereafter referred to as bronchiectasis, once thought to be an orphan disease, is being recognised with increasing frequency globally especially with the increasing availability of high resolution computed tomography [3,4,5]. Bronchiectasis is associated with irreversible bronchial dilatation in patients who suffer from productive cough, purulent sputum and recurrent infective exacerbations [6,7]. Patients experience 1.5–6.5 episodes of exacerbations per year [8], leading to increased risk of admission and readmission to hospital and potentially high healthcare costs [9]. A further study demonstrated that the prevalence of bronchiectasis increased by 8.7% per year between 2000 and 2007 [10].

Compared to other respiratory conditions, bronchiectasis has attracted relatively less attention, possibly because of lack of specific therapy for management, and the perception that severe bronchiectasis is less common [8]. While there has been increasing research looking at effects of more common respiratory conditions such as chronic obstructive pulmonary disease (COPD) and its relationship with heart attacks, strokes, cognitive function and sleep disturbances, few data are available on the relationship between bronchiectasis and certain neurological diseases and cardiovascular risk factors such as hypertension, diabetes mellitus and atrial fibrillation. Furthermore, few have looked at how a patient’s sleep and quality of life are affected in bronchiectasis who could be suffering from increased sputum production and regular nocturnal coughing, potentially leading to more arousals and impairment in daytime function.

In this review, we aim to highlight existing literature on the relationship between bronchiectasis and neurological conditions, such as stroke, cognitive function and cerebral infection, as well as discussing effects on sleep disturbances in patients with bronchiectasis.

## 2. Stroke and Its Associated Risk Factors

The pathogenesis of atherosclerosis is intimately associated with inflammation. In a review article, Libby elegantly explained the involvement of leukocytes and expression of pro-inflammatory cytokines which characterise early atherosclerosis [11]. Furthermore, the lack of inflammatory mediators halts atheroma formation in mice [12,13]. In clinical studies, systemic inflammation in conditions such as rheumatoid arthritis promote carotid intimal medial thickness plaque formation [14,15].

**Figure 1:**
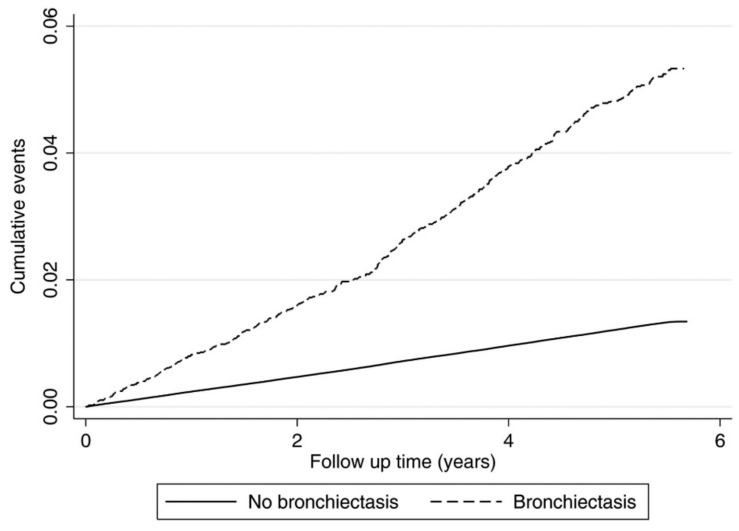
Nelson-Aalen cumulative incidence of stroke in people with bronchiectasis and those without bronchiectasis. Figure reproduced from [21].

The development of bronchiectasis is perhaps best known by the hypothesis proposed by Cole back in 1986, known as the Cole’s “vicious cycle hypothesis” [16]. In his paper, Cole proposed that an environmental insult on a background of genetic susceptibility and impaired muco-ciliary clearance result in persistence of microbes in the sino-bronchial tree and microbial colonisation. The microbial infection causes chronic inflammation resulting in tissue damage and impaired muco-ciliary motility. This in turn leads to more infection with a cycle of progressive inflammation causing lung damage [17]. Several studies have demonstrated that bronchiectasis is indeed a systemic inflammatory condition by measuring inflammatory markers such as erythrocyte sedimentation rate, white cell count, c-reactive protein, and tumor necrosis factor alpha [18,19,20]. Research on stroke and bronchiectasis is scarce. Recently, Navaratnam et al. looked at the association between bronchiectasis and cardiovascular disease [21]. Using an anonymised primary care patient electronic record database of 3,895,710 adults from 625 general practices throughout the UK, the Clinical Practice Research Datalink (CPRD), the authors found a total of 10,942 people (0.3%) had bronchiectasis, in which the majority were female (60.4%). They initially compared the relationship between coronary heart disease (CHD) and stroke in people with and without bronchiectasis, and investigated whether those with bronchiectasis were at increased risk of incident CHD and stroke events in a subsequent cohort study. Results showed significantly higher odds of coronary heart disease (OR 1.33) and stroke (OR 1.92) in those with bronchiectasis. Furthermore, patients with bronchiectasis had a 69% higher rate of first stroke than those without over a median follow up period of 5.6 years. The cumulative incidence of ischemic stroke was significantly higher in the bronchiectasis cohort than in the comparison cohort (Figure 1). They also reported an increased prevalence of risk factors for coronary artery disease and stroke in patients with bronchiectasis, namely hypertension, hyperlipidemia, diabetes and family history of cardiovascular disease. A study by Onen et al. looked at factors related to mortality in patients with bronchiectasis [22]. The study found that patients with bronchiectasis reported a higher prevalence of cardiovascular risk factors, whereby 5.1% of patients had diabetes, and 21.4% of patients had hypertension. However, the increased prevalence of cardiovascular risk factors was not significantly associated with mortality. This could be due to the small sample size of 98 patients. More recently, McDonnell and colleagues have developed a quantitative risk stratification tool, the Bronchiectasis Aetiology and Comorbidity Index (BACI), to identify patients with comorbidities accompanying bronchiectasis that are associated with a higher risk of mortality and exacerbations over the subsequent five years. Thirteen comorbidities have been identified and interestingly include ischemic heart disease, diabetes, peripheral vascular disease and cognitive impairment [23].

Using a population-based cohort study, Chen et al. examined 1295 patients from an Asian population who were newly diagnosed with bronchiectasis from year 2000 to 2008, and compared them with 6475 patients without bronchiectasis from the same cohort. The subjects were followed up to the date of ischemic stroke development, censoring for the end of 2010. This study reported an incidence rate of 9.18 per 1000 person-years of ischemic stroke in patients with bronchiectasis compared to an incidence rate of 4.66 person-years in patients without bronchiectasis [24]. The study also found that the risk of ischemic stroke in patients with bronchiectasis increased with the annual number of emergency department visits and hospitalisations, with one to three visits annually associated with a hazard ratio (HR) of 4.31, and more than three visits annually associated with a HR of 6.24. Furthermore, in patients with co-morbidities of hypertension, diabetes and atrial fibrillation (AF), the risk of ischemic stroke became *multiplicative*. For example, patients with bronchiectasis alone and diabetes alone had a HR for stroke of 2.0 and 2.20, respectively, while those patients with both bronchiectasis and diabetes had a HR of 4.42. Similar results were noted for hypertension. In patients with bronchiectasis and AF, HR for stroke was 1.88 and 1.89 respectively for each disorder alone, and 7.02 in patients with both bronchiectasis and AF.

The association between bronchiectasis and stroke is likely to be multi-factorial. Firstly, inflammation associated with bronchiectasis may promote development of atherosclerosis and risk of stroke as described above. Secondly, acute infections associated with bronchiectasis induce an acute inflammatory response and could transiently increase risk of vascular events such as stroke [25,26]. Thirdly, acute respiratory tract infections like *Chlamydia pneumoniae* and influenza infection have been directly implicated to increase the risk of stroke, although the mechanism has yet to be elucidated [27,28,29,30]. Finally, poor sleep quality is increasingly recognised as a risk factor for stroke [31,32,33]. As seen in the next section, patients with bronchiectasis suffer from sleep disturbance [6].

## 3. Sleep Disturbance

Good sleep hygiene is crucial for body and mind rejuvenation. Sleep disturbances are common in chronic lung diseases, such as COPD, idiopathic pulmonary fibrosis and cystic fibrosis, which lead to daytime fatigue, low mood and impaired quality of life [34,35,36]. While the cardinal symptom of bronchiectasis is a chronic productive cough [37], there is unfortunately limited research looking at effects of bronchiectasis on sleep.

Gao et al. looked at adults with steady state bronchiectasis and reported 56.9% of patients with bronchiectasis had sleep disturbances based on a validated questionnaire, Pittsburgh Sleep Quality Index (PSQI) (>5), compared to 28.8% in healthy subjects [6]. However, daytime sleepiness as measured using the Epworth Sleepiness Score was similar between patients with and without bronchiectasis. The major determinants associated with sleep disturbance were depression (OR 10.09), increased 24 h sputum volume (OR 2.01), and increased nocturnal cough (OR 1.89), with depression having the largest impact. This suggests that co-existing psychological morbidity can have a significant impact on sleep disturbance in patients with bronchiectasis. Other studies looking into chronic diseases have found a similar association as well, reporting sleep disorders as a core symptom of depression [38,39,40]. Therefore, treatment of depression is pertinent when addressing patients with bronchiectasis who are reporting sleep disturbances.

Another study looked at sleep quality in bronchiectasis in the pediatric population [41]. The study reported a more than twofold higher incidence of poor sleep quality in children with bronchiectasis based on the PSQI score than in children without. Furthermore, patients with bronchiectasis who suffered from nocturnal wheezing also suffered from impaired subjective sleep quality, sleep latency and higher daytime dysfunction scores compared with patients without nocturnal symptoms. There reportedly was a positive correlation between severity of bronchiectasis on high resolution computed tomography (HRCT) with sleep quality. However, Gao et al. found no positive correlation in their study of the adult population [6]. There are two multi-dimensional grading systems used for classifying the severity of bronchiectasis, the bronchiectasis severity index (BSI) and the FACED score [42]. Both these validated scores measure the extent of bronchiectasis on radiological assessment and incorporate clinical, sputum microbiology and spirometric criteria. While they are used to predict morbidity and mortality, it would be interesting to research if the severity of bronchiectasis as measured by these scales correlate with sleep disturbance, or even cognitive dysfunction and cardiovascular risk.

A novel cross-sectional study involving 49 patients with radiologically confirmed bronchiectasis formally evaluated sleep disturbance in bronchiectasis using polysomnography [43]. The study reported that 51% were male; all had a body mass index within the normal range, and a predominantly obstructive ventilatory defect on spirometry with a mean post bronchodilator forced expiratory volume in 1 s (FEV_1_) of 57.39%. Obstructive sleep apnea (OSA), defined as apnea-hypopnea index of ≥5 events per hour, was found in 41% of patients. Excessive daytime sleepiness, as measured using the Epworth Sleepiness score, was exhibited by 53% of the patients. Patients with OSA had a significantly higher prevalence of *Pseudomonas aeruginosa* colonisation (35% vs. 7.4%; *p* = 0.026). We postulate that with airflow obstruction and gas trapping, patients with bronchiectasis experience hypoxemia and hypercapnia, which consequently lead to increased arousals, similar to that reported in patients with COPD and cystic fibrosis [44,45]. More research is required to investigate the presence of sleep-disordered breathing in patients with bronchiectasis.

In our literature review, we searched for the relationship between bronchiectasis and other sleep-related disorders such as restless leg syndrome, periodic limb movement disorder, hyper-somnolence and narcolepsy but did not find any studies or case reports. These are potentially areas for future research.

## 4. Cognitive Function and Psychological Issues

Studies have shown some chronic respiratory diseases such as COPD have been associated with cognitive dysfunction [46,47,48,49]. The cognitive domains most commonly affected are memory and attention, with a reported prevalence of impairment in visuospatial memory and intermediate visual memory of 26.9% and 19.2%, respectively [50].

Few studies have looked at cognitive function in patients with bronchiectasis. One recent study compared 30 patients with stable bronchiectasis with 25 healthy volunteers, and assessed their cognition using the Wechsler Adult Intelligence Scale [51]. The study reported that patients with bronchiectasis exhibited lower verbal and performance tests and IQ scores, compared to healthy subjects. Furthermore, among subjects with bronchiectasis, those who exhibited lower cognitive ability had significantly higher depression scores and a lower oxygen saturation with both these variables significantly contributing to cognitive ability in a multivariate model.

The lower cognitive ability in bronchiectasis is likely due to several factors. Hypoxia is a probable contributory factor and has been associated with increased risk of cognitive impairment [47]. Grant et al. demonstrated that in patients with COPD, neuropsychological impairment was inversely related with PaO_2_ and resting arterial oxygen saturation [52]. Furthermore, a neuroimaging study using single photon emission computed tomography (SPECT) comparing 15 COPD patients with hypoxemia and 18 COPD patients without hypoxemia found that patients with hypoxemia had anterior cerebral hypo-perfusion on imaging, compared to non-hypoxemic patients who had comparable SPECT imaging results to normal subjects [53].

Depression likely contributes to low cognitive ability in patients with bronchiectasis similar to previous studies, including a study looking at cognitive change in 836 adults aged over 70 years, which demonstrated higher depressive symptoms were associated with poorer initial performance in processing speed, verbal fluency and episodic memory [54]. A meta-analysis by McDermott et al. reported increasing severity of depression was associated with poorer episodic memory, executive function, and processing speed [55].

The prevalence of depression in bronchiectasis patients has been reported to be from 20% to as high as 34% [56,57,58,59]. Ozgun et al. analysed 75 patients with stable bronchiectasis, and using the hospital depression anxiety scale (HAD), 15 (20%) patients showed symptoms of depression and 29 (38.7%) showed symptoms of anxiety [57]. Another study by Ozgun et al. reported depression in bronchiectasis was significantly associated with admission to an emergency department (OR 4.236), hemoptysis (OR 0.255) and living with a partner (OR 0.176). Anxiety in bronchiectasis has also been related to education level (OR 7.613), previous history of depression (OR 7.710), admission to an emergency department within the last year (OR 3.177), and living with a partner (OR 0.075) [56]. As such, depression and anxiety are intimately associated with bronchiectasis and may add to burden of disease.

## 5. Cerebral Infection

There have been several documented cases of cerebral infection in relation to bronchiectasis. There is one report of a 25-year-old male with a history of bronchiectasis for five years, with a single brain abscess in the right temporo-occipital region [60]. Patel et al. reported a case of 34 discrete brain abscesses in a patient with bronchiectasis, highlighting the potentially fatal complication of this chronic lung disease [61]. Melo et al. reported a case of a 44-year-old woman with pulmonary tuberculosis in childhood leading to extensive bilateral bronchiectasis, presenting with confusion and 6th cranial nerve palsy, eventually found to have multifocal brain abscesses [62].

The complication of brain abscesses may occur due to hematogenous spread from chronic pulmonary infections such as bronchiectasis [63]. The incidence of brain abscesses has, however, reduced substantially with the introduction of antibiotics in the current era [64].

## 6. Neurological Conditions with Aspiration

There have been studies associating bronchiectasis with some neurological conditions such as type 1 Arnold-Chiari malformation, cerebral palsy and ataxia telangiectasia (AT) [65,66,67]. The presumed pathophysiology behind this association is recurrent aspirations from the neurological condition leading to repeated chest infections, eventually causing both post-inflammatory bronchiectasis and lung parenchymal damage [67,68].

Cerebral palsy is associated with many respiratory conditions, such as asthma, recurrent pneumonia, bronchiectasis and obstructive sleep apnea [69]; the pathophysiology is multi-factorial. In cerebral palsy, many patients suffer from kyphoscoliosis, which can affect normal respiratory muscle function and decrease chest wall compliance [67]. Patients who have cerebral palsy can have severe neurocognitive impairment and have no spontaneous cough or an insufficiently forceful cough [69]. The swallowing mechanism is also often abnormal owing to dystonia and poor coordination in cerebral palsy [70]. Gastroesophageal reflux is more prevalent in children with cerebral palsy [71], which, if taking into account impaired cough and swallowing mechanism, may lead to chronic micro-aspiration, recurrent lower respiratory tract infections, ongoing airway inflammation and secondary bronchiectasis.

Ataxia telangiectasia, an autosomal recessive disorder due to a gene defect in chromosome 11q22.3, is associated with progressive lung disease, which is a major cause of morbidity and mortality in patients with AT [65]. Many patients with AT have difficulty with coordination of swallowing and may aspirate foods, liquids, and oral secretions [72,73]. In addition, neuromuscular weakness can result in decreased tidal volumes and ineffective cough, rendering AT patients unable to clear respiratory secretions effectively [74]. Many AT patients also suffer from immunodeficiency, making them more prone to protracted infections [75].

In the case report by Campisi, a 54-year-old woman with type 1 Arnold-Chiari malformation presenting with recurrent aspiration-induced pneumonia was found to have cylindrical bronchiectasis [66]. The postulated mechanism for recurrent aspiration is stretch injury to the lower cranial nerves from caudal displacement of medulla or compression of the swallowing centres in the brainstem [76]. This study also performed polysomnography to investigate daytime fatigue, which revealed central sleep apnea, again considered to be due to abnormalities in respiratory control from Arnold-Chiari malformation.

## 7. Conclusions

Bronchiectasis may be related to diseases in multiple body systems, including the neurological system, and is associated with an increased risk of stroke and cerebral infection, impaired cognitive function and impact on sleep quality in patients with bronchiectasis. Further research is required in this area, and to evaluate whether early intervention can prevent or reduce some of these potential neurological sequelae. From a clinical perspective, a holistic approach that includes an assessment of neuropsychological and sleep disorders in patients with bronchiectasis is warranted.
